# Supplementary material for: Host–microbiome coevolution can promote cooperation in a rock–paper–scissors dynamics
Source: Proc Biol Sci. 2020 Feb 12;287(1920):20192754. doi: 10.1098/rspb.2019.2754 (PMC7031668; doi:10.1098/rspb.2019.2754)
Supplement: Supplementary Information [file rspb20192754supp1.pdf]

Supplementary Information

Supplementary Note S1 The host-microbiome co-evolution model

In this section we describe the full model used for this study, and present the core equations. We model an asexual population of hosts, and assume that each host carries one of two microbe types. Microbes of type α increase the tendency of their hosts to cooperate, while microbes of type β don't affect the host behavior. In addition, we model a host gene that determines the susceptibility of the host to the microbial effect. Hosts carrying allele S are susceptible to the microbe's effect and act cooperatively when carrying microbe α. Hosts carrying allele R are resistant to the microbial effect and act selfishly at all times, but this resistance confers a fitness cost. In this section we consider a more general modeling of the resistance cost, by allowing two different resistance costs, that depend on the type of the carried microbe. We denote by $0 < \delta_\alpha, \delta_\beta < 1$ the fitness cost of resistance, for hosts carrying microbes of type α and hosts carrying microbes of type β, respectively. We thus have in our model a haploid population with four different types of hosts: $\alpha S, \alpha R, \beta S, \beta R$, defined by the combination of allele (R/S) and microbe (α/β). We assume that the cost of resistance is applied before any horizontal transfer occurs. We model the interactions payoff and horizontal transmissions as defined in the **Results** section. Note that in the main text we show results for the case of $\delta_\alpha = \delta_\beta = \delta$, and analysis of the equilibrium in that case is presented in section 3.2 of this SI.

We denote by $x_{\alpha S}, x_{\alpha R}, x_{\beta S}, x_{\beta R}$ the proportions of the host types in the current generation, and calculate $x'_{\alpha S}, x'_{\alpha R}, x'_{\beta S}, x'_{\beta R}$, the proportions of the host types in the next generation:

1. $$x'_{\alpha S} = f_1(x_{\alpha S}, x_{\alpha R}, x_{\beta S}, x_{\beta R}, c, b, \delta_\alpha, \delta_\beta, T_\alpha, T_\beta)$$
$$= \frac{1}{\bar{\omega}}\big(x_{\alpha S}^2(1+b-c) + x_{\alpha S}x_{\alpha R}(1-c) + x_{\alpha S}x_{\beta S}\big[(1-T_\beta)(1-c) + T_\alpha(1+b)\big]$$
$$+ x_{\alpha S}x_{\beta R}(1-T_\beta)(1-c) + x_{\alpha R}x_{\beta S}T_\alpha\big)$$

2. $x'_{\alpha R} = f_2(x_{\alpha S}, x_{\alpha R}, x_{\beta S}, x_{\beta R}, c, b, \delta_\alpha, \delta_\beta, T_\alpha, T_\beta)$

$$= \frac{1}{\overline{\omega}}\Big(x_{\alpha R}^2(1-\delta_\alpha) + x_{\alpha R}x_{\alpha S}(1+b-\delta_\alpha) + x_{\alpha R}x_{\beta S}(1-T_\beta)(1-\delta_\alpha) + x_{\alpha R}x_{\beta R}\left[(1-T_\beta)(1-\delta_\alpha) + T_\alpha(1-\delta_\beta)\right] + x_{\alpha S}x_{\beta R}T_\alpha(1+b-\delta_\beta)\Big)$$

3. $x'_{\beta S} = f_3(x_{\alpha S}, x_{\alpha R}, x_{\beta S}, x_{\beta R}, c, b, \delta_\alpha, \delta_\beta, T_\alpha, T_\beta)$

$$= \frac{1}{\overline{\omega}}\Big(x_{\beta S}^2 + x_{\beta S}x_{\alpha S}\left[(1-T_\alpha)(1+b) + T_\beta(1-c)\right] + x_{\beta S}x_{\alpha R}(1-T_\alpha) + x_{\beta S}x_{\beta R} + x_{\alpha S}x_{\beta R}T_\beta(1-c)\Big)$$

4. $x'_{\beta R} = f_4(x_{\alpha S}, x_{\alpha R}, x_{\beta S}, x_{\beta R}, c, b, \delta_\alpha, \delta_\beta, T_\alpha, T_\beta)$

$$= \frac{1}{\overline{\omega}}\Big(x_{\beta R}^2(1-\delta_\beta) + x_{\beta R}x_{\alpha S}(1-T_\alpha)(1+b-\delta_\beta) + x_{\beta R}x_{\beta S}(1-\delta_\beta) + x_{\beta R}x_{\alpha R}\left[T_\beta(1-\delta_\alpha) + (1-T_\alpha)(1-\delta_\beta)\right] + x_{\alpha R}x_{\beta S}T_\beta(1-\delta_\alpha)\Big)$$

where

5. $\overline{\omega} = 1 - x_{\alpha R}\delta_\alpha - x_{\beta R}\delta_\beta + x_{\alpha S}(b-c)$

Supplementary Note S2 Invasion Analysis

In order to analyze the stability of the four trivial equilibriums, where one type is at fixation and the others are extinct, we perform invasion analysis. We calculate the Jacobian of the system (f_1, f_2, f_3, f_4), by deriving the functions with respect to the variables $x_{\alpha S}, x_{\alpha R}, x_{\beta S}, x_{\beta R}$. We then calculate the eigenvalues of the Jacobian in the four trivial equilibrium points.

We find that the Jacobian matrix at the equilibrium $x_{\alpha S} = 1$ is:

6. $$J|_{x_{\alpha S}=1} = \begin{pmatrix} \frac{2+b-c}{1+b-c} & \frac{1+\delta_\alpha - c}{1+b-c} & \frac{(1-T_\beta)(1-c)+T_\alpha(1+b)}{1+b-c} & \frac{(1-T_\beta)(1-c)+\delta_\beta}{1+b-c} \\ 0 & \frac{1+b-\delta_\alpha}{1+b-c} & 0 & \frac{T_\alpha(1+b-\delta_\beta)}{1+b-c} \\ 0 & 0 & \frac{(1-T_\alpha)(1+b)+T_\beta(1-c)}{1+b-c} & \frac{T_\beta(1-c)}{1+b-c} \\ 0 & 0 & 0 & \frac{(1-T_\alpha)(1+b-\delta_\beta)}{1+b-c} \end{pmatrix}$$

And the eigen values are $\frac{1+b-\delta_\alpha}{1+b-c}$, $\frac{(1-T_\alpha)(1+b)+T_\beta(1-c)}{1+b-c}$ and $\frac{(1-T_\alpha)(1+b-\delta_\beta)}{1+b-c}$.

Thus, when αS hosts are at fixation:

- If $\frac{1+b-\delta_\alpha}{1+b-c} > 1$ (namely, $\delta_\alpha < c$) then αR hosts can invade the population
- If $\frac{(1-T_\alpha)(1+b)+T_\beta(1-c)}{1+b-c} > 1$ (namely, $\frac{b}{c} < \frac{1-T_\beta}{T_\alpha} + \frac{T_\beta - T_\alpha}{T_\alpha c}$) then βS hosts can invade the population
- If $\frac{(1-T_\alpha)(1+b-\delta_\beta)}{1+b-c} > 1$ then βR hosts can invade the population

By changing the order of the functions and the order of the derivations to f_2, f_1, f_4, f_3 and $x_{\alpha R}, x_{\alpha S}, x_{\beta R}, x_{\beta S}$ respectively, we find that the Jacobian matrix at the equilibrium $x_{\alpha R} = 1$ is:

7. $$J|_{x_{\alpha R}=1} = \begin{pmatrix} \frac{2-\delta_\alpha}{1-\delta_\alpha} & \frac{1+c-\delta_\alpha}{1-\delta_\alpha} & \frac{T_\alpha(1-\delta_\beta)+(1-T_\beta)(1-\delta_\alpha)+\delta_\beta}{1-\delta_\alpha} & 1-T_\beta \\ 0 & \frac{1-c}{1-\delta_\alpha} & 0 & \frac{T_\alpha}{1-\delta_\alpha} \\ 0 & 0 & \frac{T_\beta(1-\delta_\alpha)+\ (1-T_\alpha)(1-\delta_\beta)}{1-\delta_\alpha} & T_\beta \\ 0 & 0 & 0 & \frac{1-T_\alpha}{1-\delta_\alpha} \end{pmatrix}$$

And the eigen values are $\boldsymbol{\frac{1-c}{1-\delta_\alpha}}$, $\boldsymbol{\frac{T_\beta(1-\delta_\alpha)+\ (1-T_\alpha)(1-\delta_\beta)}{1-\delta_\alpha}}$ and $\boldsymbol{\frac{1-T_\alpha}{1-\delta_\alpha}}$.

Thus, when αR hosts are at fixation:

- If $\frac{1-c}{1-\delta_\alpha} > 1$ (namely, $c < \delta_\alpha$) then αS hosts can invade the population
- f $\frac{T_\beta(1-\delta_\alpha)+\ (1-T_\alpha)(1-\delta_\beta)}{1-\delta_\alpha} > 1$ then βR hosts can invade the population
- If $\frac{1-T_\alpha}{1-\delta_\alpha} > 1$ (namely, $T_\alpha < \delta_\alpha$) then βS hosts can invade the population

By changing the order of the functions and the order of the derivations to f_3, f_4, f_1, f_2 and $x_{\beta S}, x_{\beta R}, x_{\alpha S}, x_{\alpha R}$ respectively, we find that the Jacobian matrix at the equilibrium $x_{\beta S} = 1$ is:

8. $$J|_{x_{\beta S}=1} = \begin{pmatrix} 2 & 1+\delta_\beta & 1+c+T_\beta(1-c)-T_\alpha(1+b) & 1+\delta_\alpha-T_\alpha \\ 0 & 1-\delta_\beta & 0 & T_\beta(1-\delta_\alpha) \\ 0 & 0 & T_\alpha(1+b)+(1-T_\beta)(1-c) & T_\alpha \\ 0 & 0 & 0 & (1-T_\beta)(1-\delta_\alpha) \end{pmatrix}$$

and the eigen values for invasion are: $\boldsymbol{1-\delta_\beta}$, $\boldsymbol{T_\alpha(1+b)+(1-T_\beta)(1-c)}$ and $\boldsymbol{(1-T_\beta)(1-\delta_\alpha)}$.

Thus, when βS hosts are at fixation:

- If $1-\delta_\beta > 1$ (namely, $\delta_\beta < 0$) then βR hosts can invade the population
- If $T_\alpha(1+b)+(1-T_\beta)(1-c) > 1$ (namely, $\frac{b}{c} > \frac{1-T_\beta}{T_\alpha} + \frac{T_\beta-T_\alpha}{T_\alpha c}$) then αS hosts can invade the population
- If $(1-T_\beta)(1-\delta_\alpha) > 1$ then αR hosts can invade the population.

By changing the order of the functions and the order of the derivations to f_4, f_3, f_2, f_1 and $x_{\beta R}, x_{\beta S}, x_{\alpha R}, x_{\alpha S}$ respectively, we find that the Jacobian matrix at the equilibrium $x_{\beta R} = 1$ is:

9. $$J|_{x_{\beta R}=1} = \begin{pmatrix} \frac{2-\delta_\beta}{1-\delta_\beta} & 1 & \frac{T_\beta(1-\delta_\alpha)+(1-T_\alpha)(1-\delta_\beta)+\delta_\alpha}{1-\delta_\beta} & \frac{c-b+(1-T_\alpha)(1+b-\delta_\beta)}{1-\delta_\beta} \\ 0 & \frac{1}{1-\delta_\beta} & 0 & \frac{T_\beta(1-c)}{1-\delta_\beta} \\ 0 & 0 & \frac{T_\alpha(1-\delta_\beta)+(1-T_\beta)(1-\delta_\alpha)}{1-\delta_\beta} & \frac{T_\alpha(1+b-\delta_\beta)}{1-\delta_\beta} \\ 0 & 0 & 0 & \frac{(1-T_\beta)(1-c)}{1-\delta_\beta} \end{pmatrix}$$

and the eigen values are: $\frac{\mathbf{1}}{\mathbf{1-\delta_\beta}}$, $\frac{\mathbf{T_\alpha(1-\delta_\beta)+(1-T_\beta)(1-\delta_\alpha)}}{\mathbf{1-\delta_\beta}}$ and $\frac{\mathbf{(1-T_\beta)(1-c)}}{\mathbf{1-\delta_\beta}}$.

Thus, when βR hosts are at fixation:

- If $\frac{\mathbf{1}}{\mathbf{1-\delta_\beta}} > 1$ (namely, $\delta_\beta > 0$) then βS hosts can invade the population
- If $\frac{\mathbf{T_\alpha(1-\delta_\beta)+(1-T_\beta)(1-\delta_\alpha)}}{\mathbf{1-\delta_\beta}} > 1$, then αR hosts can invade the population
- If $\frac{\mathbf{(1-T_\beta)(1-c)}}{\mathbf{1-\delta_\beta}} > 1$ (namely, $\delta_\beta - c > T_\beta(1-c)$) then αS hosts can invade the population.

Altogether, we obtained 12 conditions for invasion of each host type, to a population dominated by another host type.

- We first note that fixation of βS is stable unless: $\delta_\beta < 0, \frac{b}{c} > \frac{1-T_\beta}{T_\alpha} + \frac{T_\beta - T_\alpha}{T_\alpha c}$, or $(1-T_\beta)(1-\delta_\alpha) > 1$. Since we focus on $0 < T_\beta, \delta_\alpha < 1$, only the second condition can disrupt the stability of βS fixation. Thus fixation of βS is stable unless $\frac{b}{c} > \frac{1-T_\beta}{T_\alpha} + \frac{T_\beta - T_\alpha}{T_\alpha c}$ (condition 1 of the main text). In addition we note that βS hosts can invade a βR-population as long as $\delta_\beta > 0$.

- Second, we note that $\frac{b}{c} > \frac{1-T_\beta}{T_\alpha} + \frac{T_\beta - T_\alpha}{T_\alpha c}$ and $\frac{(1-T_\alpha)(1+b-\delta_\beta)}{1+b-c} > 1$ contradict each other. Note that the first term can be rewritten as $T_\alpha b > c(1-T_\beta) + (T_\beta - T_\alpha)$ while the second term can be rewritten as $T_\alpha b < [c(1-T_\beta) + (T_\beta - T_\alpha)] - [T_\beta(1-c) + \delta_\beta(1-T_\alpha)]$. Thus, if αS hosts can invade βS population, then βR hosts cannot invade αS population.
- Lastly, we note that if $\frac{(\mathbf{1}-\boldsymbol{T_\beta})(\mathbf{1}-\boldsymbol{c})}{\mathbf{1}-\boldsymbol{\delta_\beta}} > 1$, then c must be smaller than δ_β. If in addition $\delta_\alpha < c$, then altogether we get that $\delta_\alpha < c < \delta_\beta$ – a scenario which we neglect, as we focus on cases where the resistance cost in the presence of manipulating microbe is equal or higher to the resistance cost in the absence of this microbe.

To conclude, we find only one subset of conditions whose intersection is not empty, maintaining $0 < \delta_\beta < \delta_\alpha$, $0 < b, c, T_\alpha, T_\beta$, that allows the invasion of αS to a population dominated by another host type, and where none of the fixation points are stable. These conditions yield a rock-paper-scissor dynamic where each of the four host types can be invaded by other host type, and intermediate levels of cooperation can be maintained.
The conditions are:

$10a.\ \frac{b}{c} > \frac{1-T_\beta}{T_\alpha} + \frac{T_\beta - T_\alpha}{T_\alpha c}$ (same as (2.1) in the main text)

$10b.\ \delta_\alpha < c$

$10c.\ \delta_\beta > 0$

$$10d.\ T_\alpha < \max\left(\delta_\alpha, T_\beta + \frac{\delta_\alpha - \delta_\beta}{1-\delta_\beta}(1-T_\beta)\right)$$

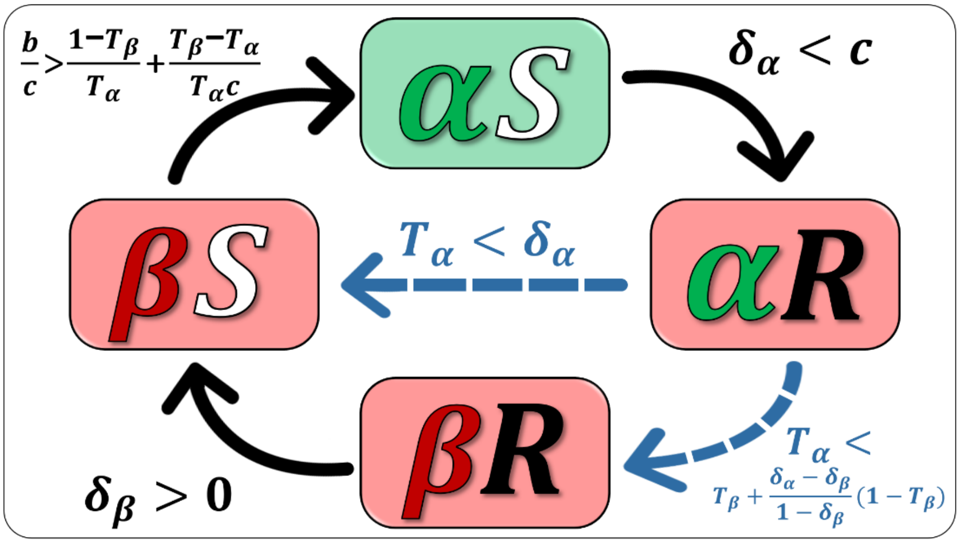

Figure S1. Invasion dynamics based on the stability analysis performed in this section. Illustrated are the set of conditions that maintain $0 < \delta_\beta, \delta_\alpha < c$, and allow a rock-paper-scissor dynamic. Note that when setting $\delta_\alpha = \delta_\beta = \delta$ we get the same dynamic as illustrated in figure 2c in the main text. We also note that $T_\beta + \frac{\delta_\alpha - \delta_\beta}{1-\delta_\beta}(1 - T_\beta) > T_\beta$ for $\delta_\alpha > \delta_\beta$, and thus the condition for $\alpha R \rightarrow \beta R$ is even milder than the one presented in the main text for $\delta_\alpha = \delta_\beta = \delta$.

When (10a-d) apply, there is no polymorphic equilibrium that involves two host types that share the same allele or microbe type. In addition, no polymorphic equilibrium that involves exactly two host types that carry different alleles and microbes can exist since horizontal transmission of the microbes will lead to generation of the other two host types as well. Similarly with three host types – no stable equilibrium can involve only three host types, since horizontal transmission will lead to the generation of the fourth host type. We thus conclude that when (10a-d) are maintained, there is no stable polymorphism on the boundaries of the 4-D standard simplex, hence the system must maintain all four host types. We find that when (10a-d) are maintained indeed the population either reach a stable polymorphic equilibrium, or oscillate chaotically.

Supplementary Note S3 Equilibrium analysis

3.1 General case of $\delta_\alpha \neq \delta_\beta$

We study the non-trivial equilibria of the system, where $x_i' = x_i$ for all $i \in \{\alpha S, \alpha R, \beta S, \beta R\}$. We begin by analyzing the equilibrium with respect to the alleles, where $x_{\alpha S}' + x_{\beta S}' = x_{\alpha S} + x_{\beta S}$ (based on equations 1 and 3). We denote $x_S = x_{\alpha S} + x_{\beta S}$, $x_R = x_{\alpha R} + x_{\beta R}$ and find that the proportion of allele S can increase from one generation to the next only when:

$$11.\quad \frac{x_{\alpha S}}{x_S} < \frac{\delta_\beta}{c} + \frac{\delta_\alpha - \delta_\beta}{c} \cdot \frac{x_{\alpha R}}{x_R}$$

and that a non-trivial equilibrium must satisfy:

$$12.\quad \frac{x_{\alpha S}}{x_S} = \frac{\delta_\beta}{c} + \frac{\delta_\alpha - \delta_\beta}{c} \cdot \frac{x_{\alpha R}}{x_R}$$

Combining (12) and (1-5), and using a numeric solver we were able to find the polymorphic equilibrium (when such existed). The analysis showed that cooperation can evolve and be maintained even when $\delta_\beta < \delta_\alpha < c$.

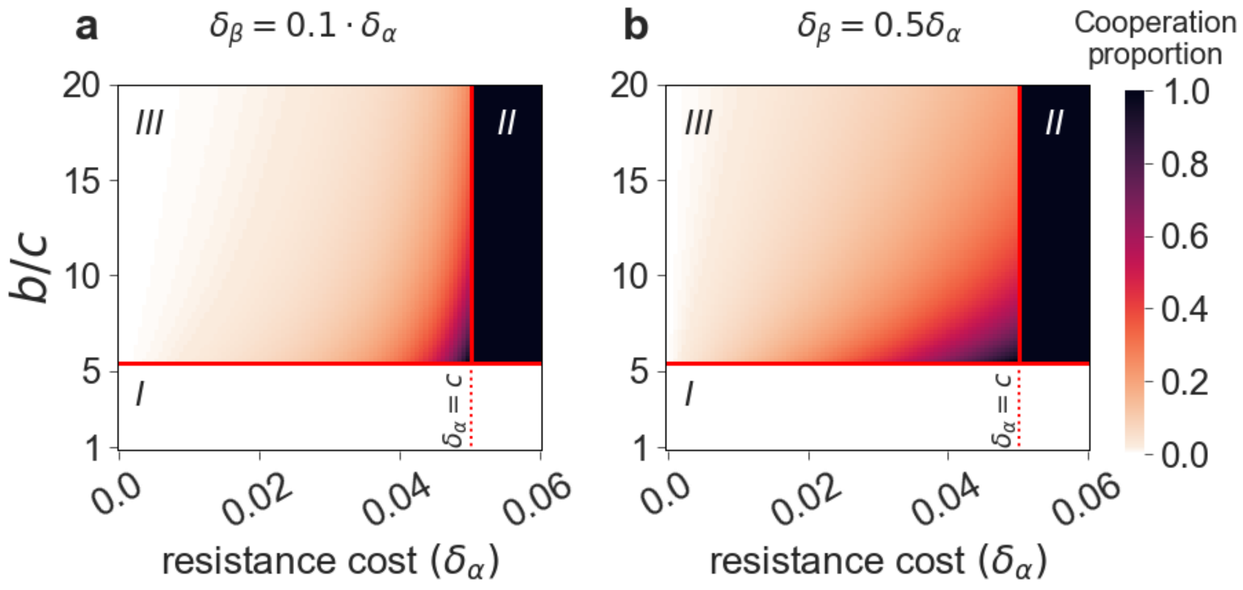

Figure S2. Cooperation can be maintained also when $\boldsymbol{\delta_\beta < \delta_\alpha}$. We plot (color coded) the expected proportion of cooperative hosts (αS) at equilibrium, as a function of b/c (y-axis) and of δ_α (x-axis) for $\delta_\beta = 0.1\delta_\alpha$ **(a)** and $\delta_\beta = 0.5\delta_\alpha$ **(b)**. Cooperation goes extinct when below the horizontal dashed line representing condition ***(2.1)*** of the main text (area ***I***, white). Above that threshold, cooperation can either go to fixation (when $\delta_\alpha > c$, area ***II***, black), or be maintained at intermediate levels (when $\delta_\alpha < c$, area ***III***). In the latter case, the proportion of cooperators increases with δ_α. We see that cooperation can be maintained even when $\delta_\beta < \delta_\alpha$, although the proportion of cooperators is lower for smaller values of $\delta_\beta/\delta_\alpha$ (compare Figure 2a from the main text, where $\delta_\alpha = \delta_\beta$ to this figure). $c = 0.05, T_\beta = 0.25, T_\alpha = 0.9 \cdot T_\beta$

3.2 Special case of $\boldsymbol{\delta_\alpha = \delta_\beta = \delta}$

We focused on the case where the cost of resistance is independent of the microbe the host carries, namely $\delta_\alpha = \delta_\beta = \delta$. In this case equation (12) becomes:

$$13.\ \frac{x_{\alpha S}}{x_S} = \frac{\delta}{c}$$

A few insights arise from this equation. First, we can see that if $\delta > c$, the equilibrium cannot exist, since $x_{\alpha S} \leq x_S$ by definition. Second, this equation reveals that the proportion of cooperators at the equilibrium, is bounded by δ/c. We further find that the proportion of allele S can increase from one generation to the next only when (same as condition (2.2) in the main text):

$$14.\ \frac{x_{\alpha S}}{x_S} < \frac{\delta}{c}$$

This means that when $\delta > c$, condition (14) applies for all $x_S > 0$ and therefore the proportion of allele S will increase from one generation to the next until it reaches fixation. After S reaches fixation, the dynamic is determined according to *(2.1)* of the main text: if the condition is maintained αS hosts will fixate, and otherwise, βS hosts will fixate.

Using (13) and (1-5) we were able to find analytically two non-trivial equilibria of the system, and derive the exact solutions. We do not present here the exact expressions, as they are too long. Based on this analysis we generated figure 2 in the main text.

We continued by analyzing the validity of the non-trivial equilibria, namely that the proportions of each the four host types at equilibrium is positive and smaller than 1. We first note that when $\delta_\alpha = \delta_\beta = \delta$, conditions (10a-d) are simplified:

$15a.\ \frac{b}{c} > \frac{1-T_\beta}{T_\alpha} + \frac{T_\beta - T_\alpha}{T_\alpha c}$ (same as (2.1) in the main text)

$15b.\ \delta < c$

$15c.\ \delta > 0$

$15d.\ T_\alpha < \max(\delta, T_\beta)$

For any parameter set that maintain (15a-d) and that we've investigated, including the ones presented in figure 2 in the main text, only one polymorphic equilibrium was found. Screening more than 10^9 parameter sets confirmed this finding. Since when (15a-d) are maintained the boundaries of the simplex are not stable (as explained in SI2), the system can either converge to the polymorphic equilibrium (as shown in Figures 3a,b in the main text), or oscillate chaotically around the equilibrium (as shown in Figures 3c,d in the main text). In any case, cooperation can evolve and be maintained at intermediate level.

We also found that there are some parameter sets that do not maintain (15a) or (15d), but still allow the existence of polymorphic equilibrium. Although we note, that the stability analysis shows that if one of the conditions (15a-d) is not maintained, then at least one of the trivial equilibria, is stable. In this case polymorphism cannot be globally stable, and if the system gets close enough to a stable trivial equilibrium it will get attracted and move towards it and remain there. When (15a-c) are maintained, but (15d) isn't (thus $T_\alpha > \max(\delta, T_\beta)$), there are some parameter sets for which equilibrium exists, but the dynamic depends on the initial proportions of the different host types. In this regime fixation of αR hosts is a stable equilibrium, and thus

even though polymorphism can exist, it cannot be globally stable. Namely, some initial conditions of the population composition lead to polymorphism, while others lead to the fixation of αR hosts. When (15a) is not maintained we find that there are two types of dynamics. If (15d) is maintained, then fixation of βS host is a stable equilibrium and it is the only one among the trivial equilibria. In this case the system drives the population towards fixation of βS hosts (verified numerically over more than 10^9 parameter sets). If on the other hand both (15a) and (15d) are not maintained, there are two stable trivial equilibria in the system – the fixation of βS hosts and the fixation of αR hosts. In this range of parameters there is also a polymorphic equilibrium. Nevertheless, in any parameter set that we've investigated, stability analysis revealed that the polymorphic equilibrium is unstable.

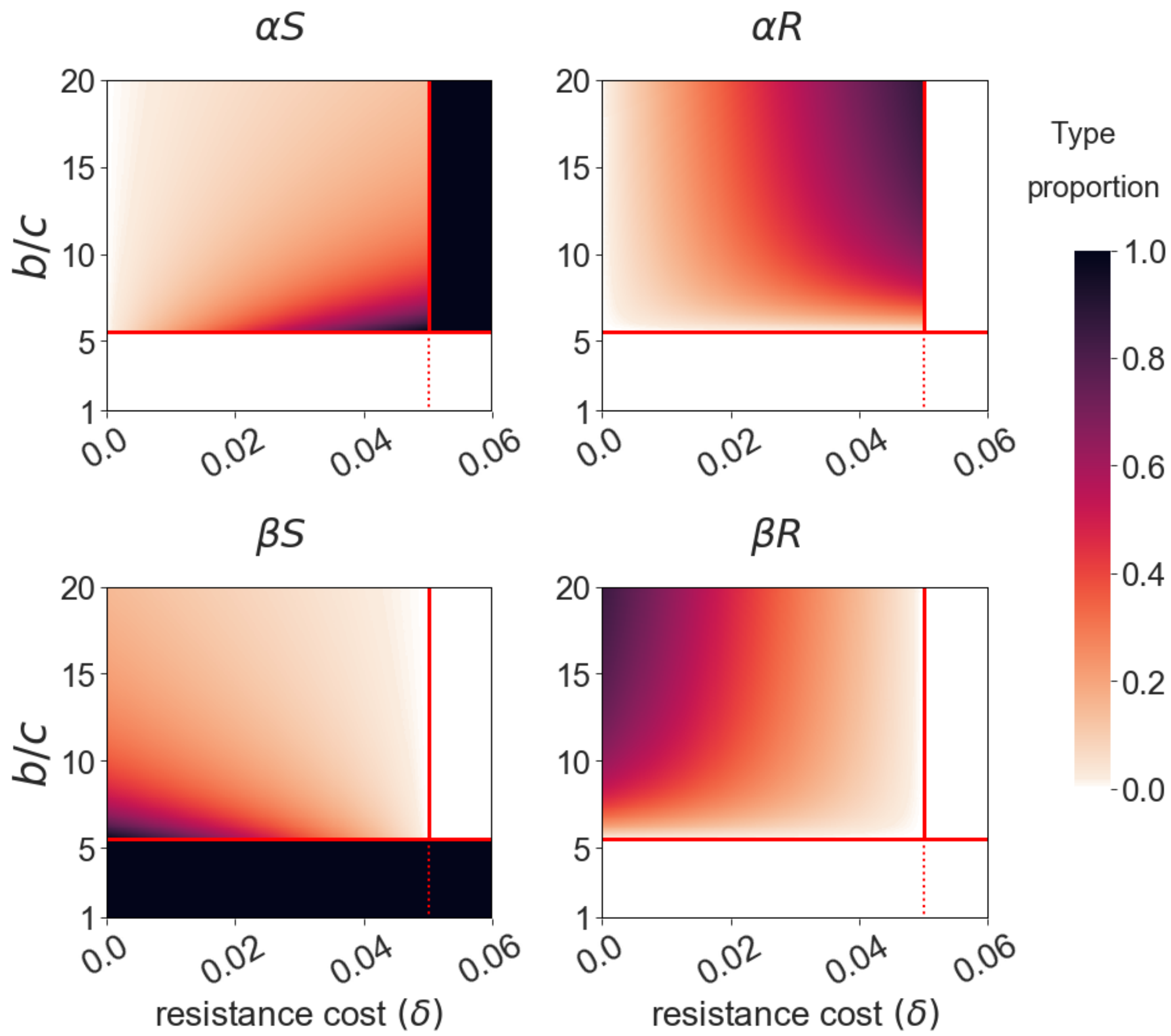

Figure S3. The expected proportions of the four different host types in the population. We plot the expected proportions of the four different host types as a function of the b/c (y-axis) and of δ (x-axis) for $c = 0.05, T_\beta = 0.25, T_\alpha = 0.9 \cdot T_\beta$. The upper-left panel is identical to figure 2b, while the other panels present the expected proportions of the three other host types.

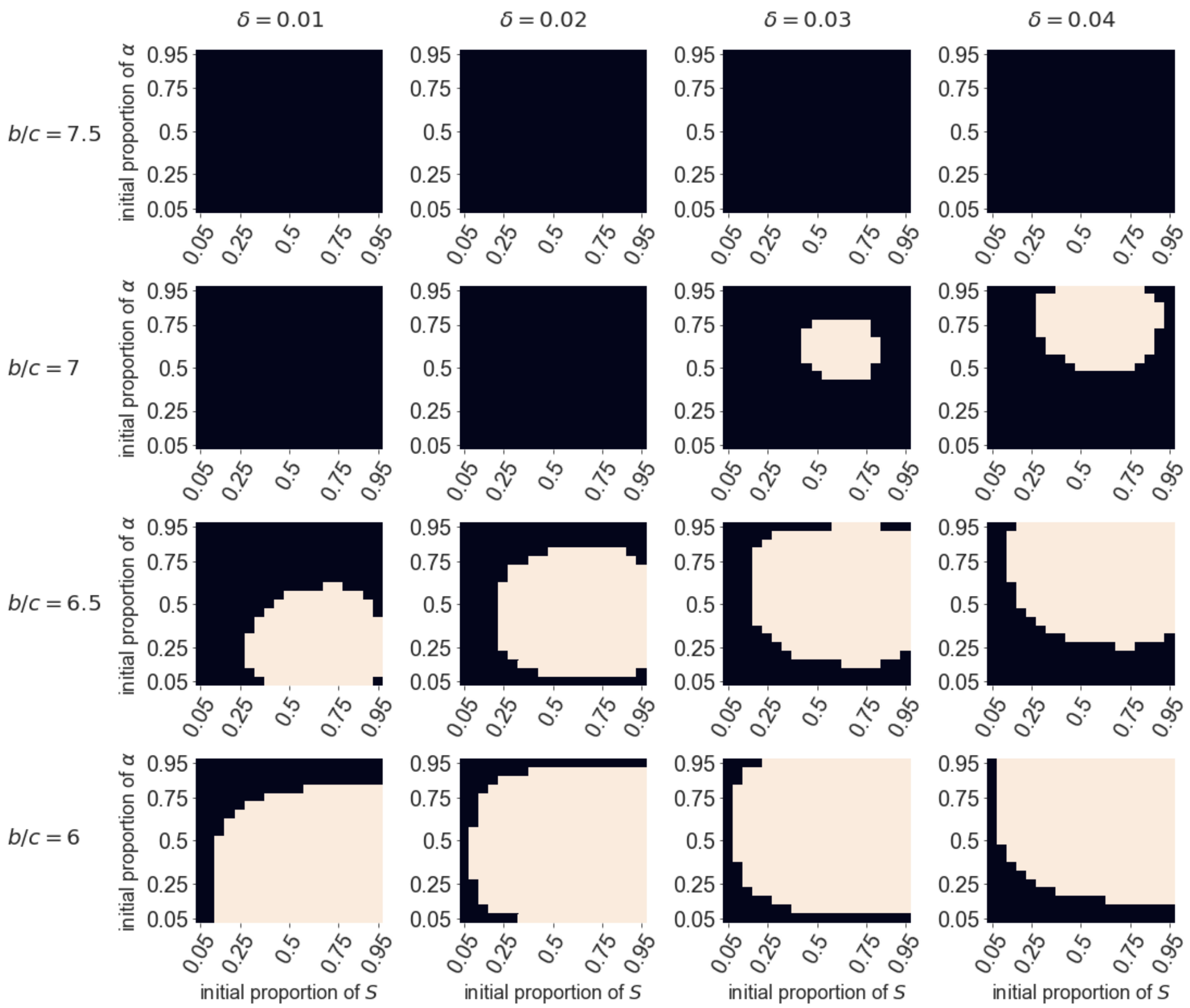

Figure S4. Initial composition of the population and the different model parameters determine whether the oscillations converge or diverge. We survey different initial proportions of microbe α (p_α) and allele (p_S), and for each p_α, p_S combination we initialize a population with the following $\alpha S, \alpha R, \beta S, \beta R$ proportions: $p_\alpha \cdot p_S, p_\alpha \cdot (1 - p_S), (1 - p_\alpha) \cdot p_S, (1 - p_\alpha) \cdot (1 - p_S)$. We then numerically iterate equations 1-5 until the population reaches equilibrium or chaotic oscillations. We plot the state to which the population was driven: convergence to equilibrium (off-white) or chaotic oscillations (black), as a function of different p_α, p_S values, and for several b/c and δ values. $T_\beta = 0.25, T_\alpha = 0.9 \cdot T_\beta, c = 0.05$.

Supplementary Note S4 Stochastic Simulations

4.1 Stochastic simulation workflow

Fully-mixed population. We programmed an agent-based simulation, where we follow $N = 10{,}000$ hosts. Each host is defined by the allele (S/R) and microbe (α/β) it carries. Each generation, the hosts are randomly divided to interacting couples K times. During an interaction, hosts that carry microbe α and allele S cooperate: they pay a fitness cost, c, and their partner receives a fitness benefit, b, regardless of its microbe-allele combination. Hosts with microbe-allele combination other than αS, don't cooperate, and thus do not pay a fitness cost of c. In addition, in each interaction the microbes can be transmitted between interacting host, with probabilities T_α, T_β. Transmission and establishment of one microbe is independent of the other microbe, and when both occur, they occur simultaneously. During an interaction, the hosts behave according to the allele and microbes they carried right before the interaction, and if horizontal transmission occurs, the effect of the new microbes on the host behavior begins right after the current interaction. The fitness of each host is determined as follows: $= 1 - \delta I_R + b \cdot n_b - c \cdot n_c$, where I_R is an indicator that equals 1 if the host carries allele R and 0 otherwise, n_b is the number of times the host interacted with a cooperator, and n_c is the number of interactions in which the host cooperated. Note that when considering multiple interactions per generation, a host can change its behavior from one interaction to the other, due to horizontal transmission of the microbes. For example, a α-carrying host will cooperate during an interaction with a β-carrying host, and it can also get infected by its β microbes. In this case, on its next interaction, this host would not cooperate, as it now carries microbes of type β. After all interactions take place, reproduction occurs. The next generation hosts are generated by considering the offspring as copies of the parent generation, and by choosing 10,000 hosts from the parent generation with a multinomial distribution, and with replacement (a parent can have more than one offspring). The probability to choose host j is its fitness divided by the sum of all the hosts' fitness: $\frac{\omega_j}{\sum_{i=1}^{N} \omega_i}$.

Spatially-structured population. For the spatially-structured scenario we programmed an agent-based simulation where we consider a 2D 100×100 lattice, where each site is inhabited by one host. Each host is defined by the allele (S/R) and microbe (α/β) it carries. Cooperation and horizontal transmissions are defined similarly to the fully-mixed case, but differently from the fully-mixed model, the interactions are local. Since there is no simple procedure for randomly dividing the entire population into interacting couples, where each couple includes two neighbors, we perform the following procedure: at each generation, the population undergoes K rounds of interactions. At each round, each host (drawn from the lattice in a random order) has a probability of p_i to initiate an interaction with a randomly drawn neighbor host, where a neighbor is defined as any host from the 8 adjacent sites (or less if close to the edge of the lattice). Thus, when setting $p_i = 1/2$ and $K = 1$, the expected number of interactions per generation each host takes part in equals to 1 in the interior of the lattice (but note that the actual number of interactions per host per generation can vary, and could also be zero). Note that there is no difference between initiating an interaction and being selected for interaction, as in both cases the two individuals play the symmetric prisoner's dilemma. The interactions, payoffs, horizontal transmissions and fitness calculations are modeled similarly to the fully-mixed model. After all interactions take place, reproduction occurs. A new lattice grid of the same size is formed. Every site in the new lattice is inhabited by a replicate (apart from mutations and imperfect vertical transmission) of a host from the neighborhood of that site in the original lattice, chosen randomly with probabilities proportional to the hosts' fitness.

Stopping criteria of the simulation. When simulating populations without mutations, we let the simulation run until either one host type reaches fixation, or 5,000 generations. For simulations with mutations, we simply let the simulation run for 5,000 generations. We also examined the effect of prolonging the simulation time to 10,000 generations, and found very similar results, except in the fully-mixed population without mutations, where the range of parameters allowing the maintenance of cooperation narrowed, as expected (see Figure S8).

4.2 mutations and imperfect vertical transmission

Mutations. We investigate the effects of mutations both in fully-mixed and spatially-structured populations. The mutations were modeled as a change in the allele or microbial population of an offspring host, relative to its parent. In the main text we show results of simulations with mutation rates of $\mu = 10^{-4}$ in all directions, namely equal probabilities for $\alpha \to \beta, \beta \to \alpha, S \to R, R \to S$ mutations. In the stochastic simulations we examine populations of size $N = 10{,}000$, hence $\mu N = 1$. We also examined mutation rates of 10^{-3} and 10^{-5} and found qualitatively similar results (see Figure S6).

Imperfect vertical transmission. We modeled imperfect vertical transmission similarly to [Lewin-Epstein et. al. 2017; see also SI]: during reproduction, with probability ρ, the offspring inherits its parent microbes, and with probability $1 - \rho$ it inherits microbes from a random host from the parent neighborhood (in a fully-mixed population the parent neighborhood includes the entire parent population).

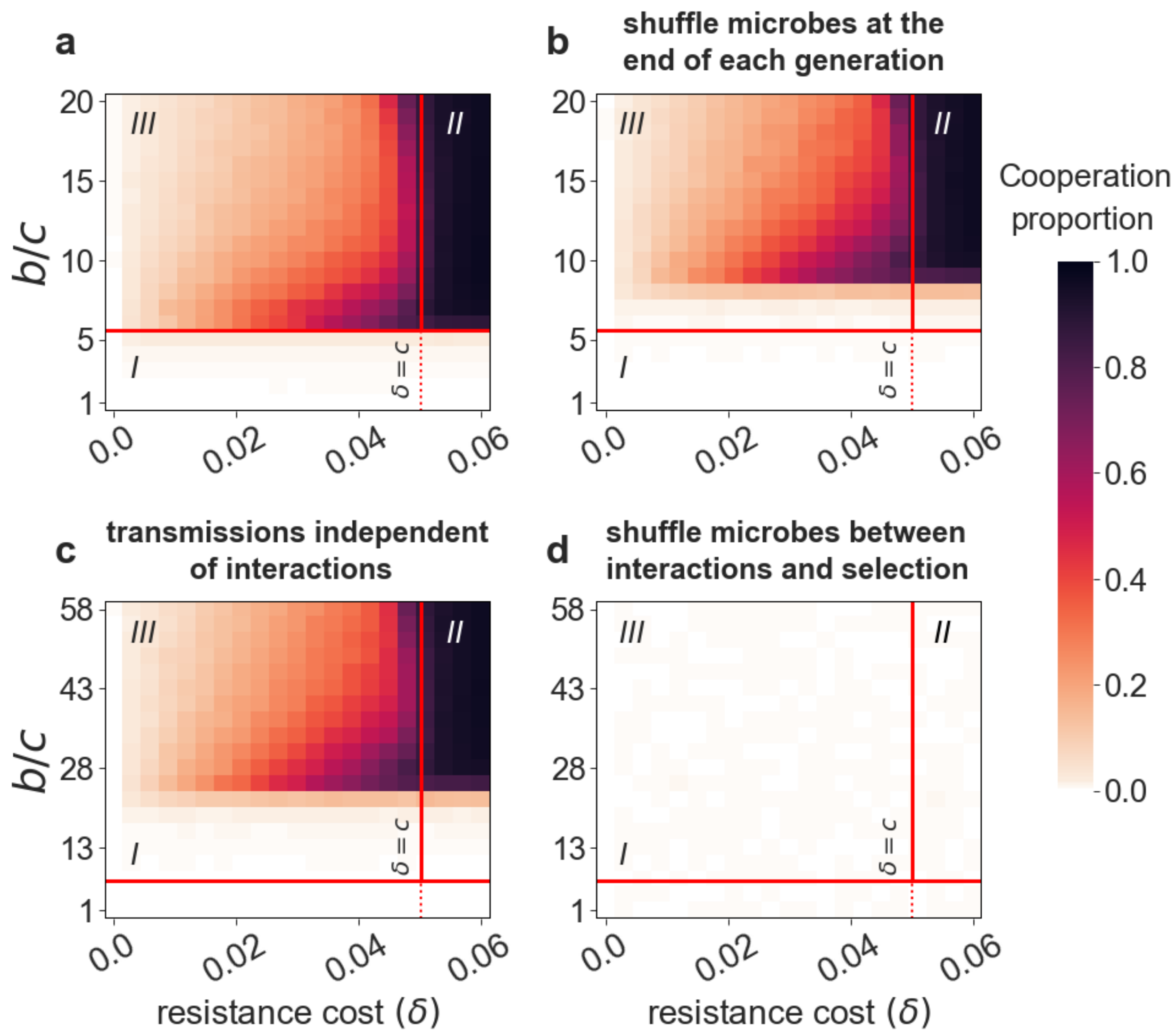

Figure S5. The effects of microbial shuffling on the evolution of cooperation. We plot the proportion of cooperators in four different settings of spatially-structured populations: identical to the workflow described in the main text (a; this panel is identical to figure 4d in the main text); shuffling of the microbes across the entire lattice at the end of each generation (b); behavioral interactions are independent of horizontal transmissions (when host initiates an interaction it first chooses a random neighbor with whom it plays the prisoners' dilemma, and then it chooses another random neighbor with whom it can exchange microbes [according to T_α and T_β]) (c); and shuffling of the microbes across the entire lattice each generation between the interactions and the selection (d). The horizontal red lines represent the analytic b/c threshold shown in inequality (2.1) in the main text. In the main text we showed that microbe-induced cooperation can evolve even without the act of kin selection among the hosts, as it succeeds in fully-mixed populations (Figure 4a and c). Introducing spatial structure allows kin selection to act also on the hosts' level, increasing the expected proportions of cooperators (a). Shuffling the microbes across all hosts at the end of each generation (b) allows kin selection among the microbes, but breaks the associations between α

and S in the next generation. Similarly to imperfect vertical transmission, this results in increasing the threshold for the evolution of cooperation. Separating between the behavioral interaction and the horizontal transmission of the microbes (c) increases the b/c threshold even more. However, since the interactions and the transmissions occur among neighbors, cooperation can still evolve above the threshold as there is still a reasonable chance ($\geq 1/8$) to choose the same host for both behavioral and horizontal transmission interactions. Finally, shuffling the microbes across all hosts after interaction and before selection breaks the linkage between the fitness change due to the interactions and the horizontal transmission of the microbes, resulting in elimination of cooperation. Panel (a) is identical to figure 4d in the main text. In all panels we use $T_\beta = 0.25, T_\alpha = 0.9T_\beta, c = 0.05, \mu = 10^{-4}$, one interaction per host per generation, and perfect vertical transmission. All simulations were initialized with equal proportions of the four host types. Each value in panels (b) and (c) is based on 100 simulation runs, while in panel (d) each value is based on 10 simulation runs.

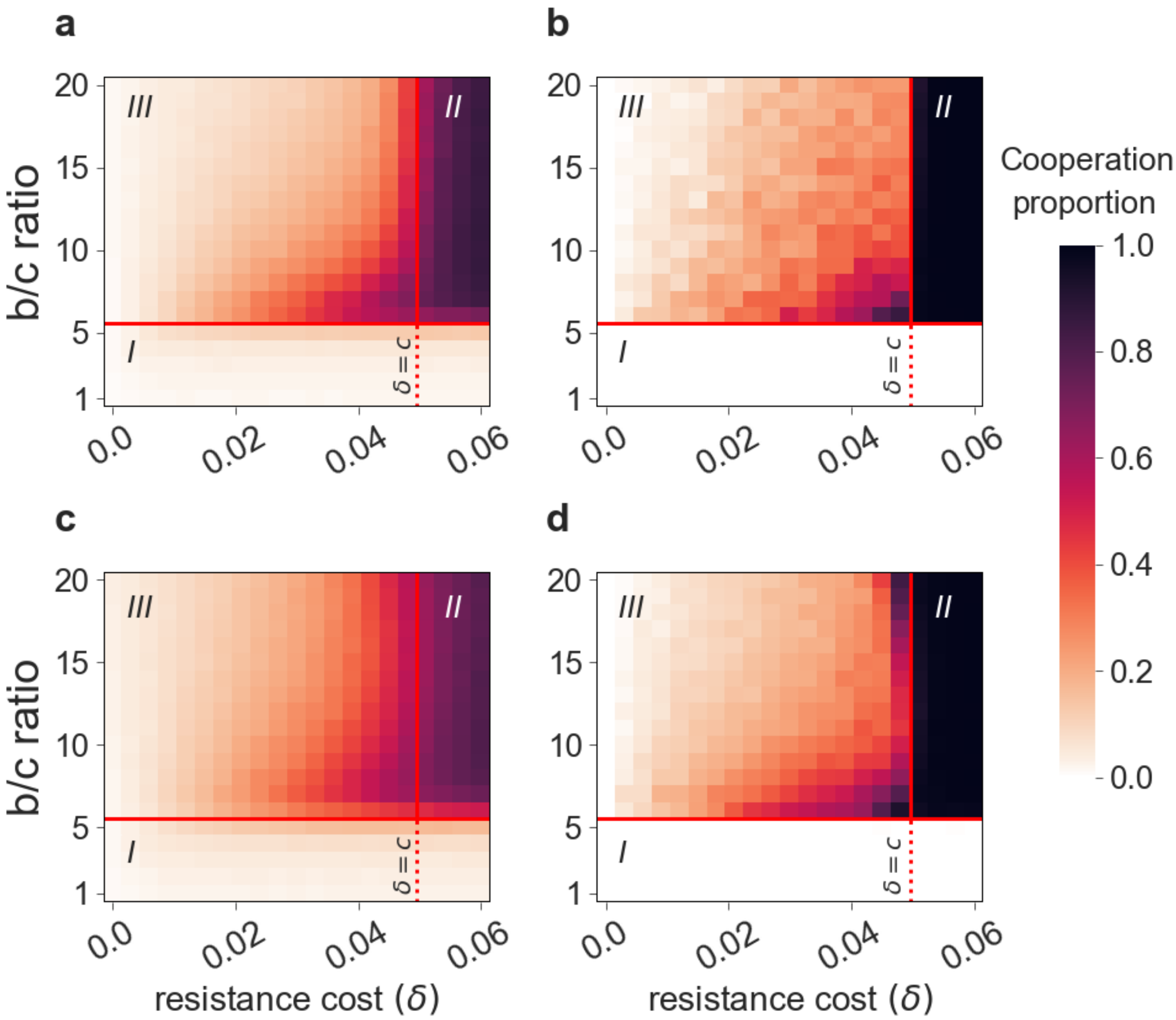

Figure S6. Cooperation is maintained under varied mutation rates. Similarly to figure 4 in the main text, the proportion of cooperators after up to 5,000 generations is plotted as a function of the b/c ratio on the y-axis and δ on the x-axis (see SI4.1 for stopping criteria). The color of each site represents the average of 100 stochastic simulation runs. Panels **(a)** and **(b)** show the results of fully-mixed populations, while panels **(c)** and **(d)** show results of spatially-structured populations. The horizontal red lines represent the analytic b/c threshold shown in inequality (2.1) in the main text. For panels **(a)** and **(c)** we set mutation rate $\mu = 10^{-3}$ in all directions ($\alpha \leftrightarrow \beta$ *and* $S \leftrightarrow R$), while for panels **(b)** and **(d)** we used $\mu = 10^{-5}$ in all directions. Note that in panels **(a)** and **(c)**, when $\mu = 10^{-3}$, limited cooperation is maintained even below the b/c threshold derived from condition (2.1) of the main text, due to mutation-selection balance. Simulation parameters: $T_\beta = 0.25, T_\alpha = 0.9T_\beta, c = 0.05$. All simulations were initialized with equal proportions of the four host types.

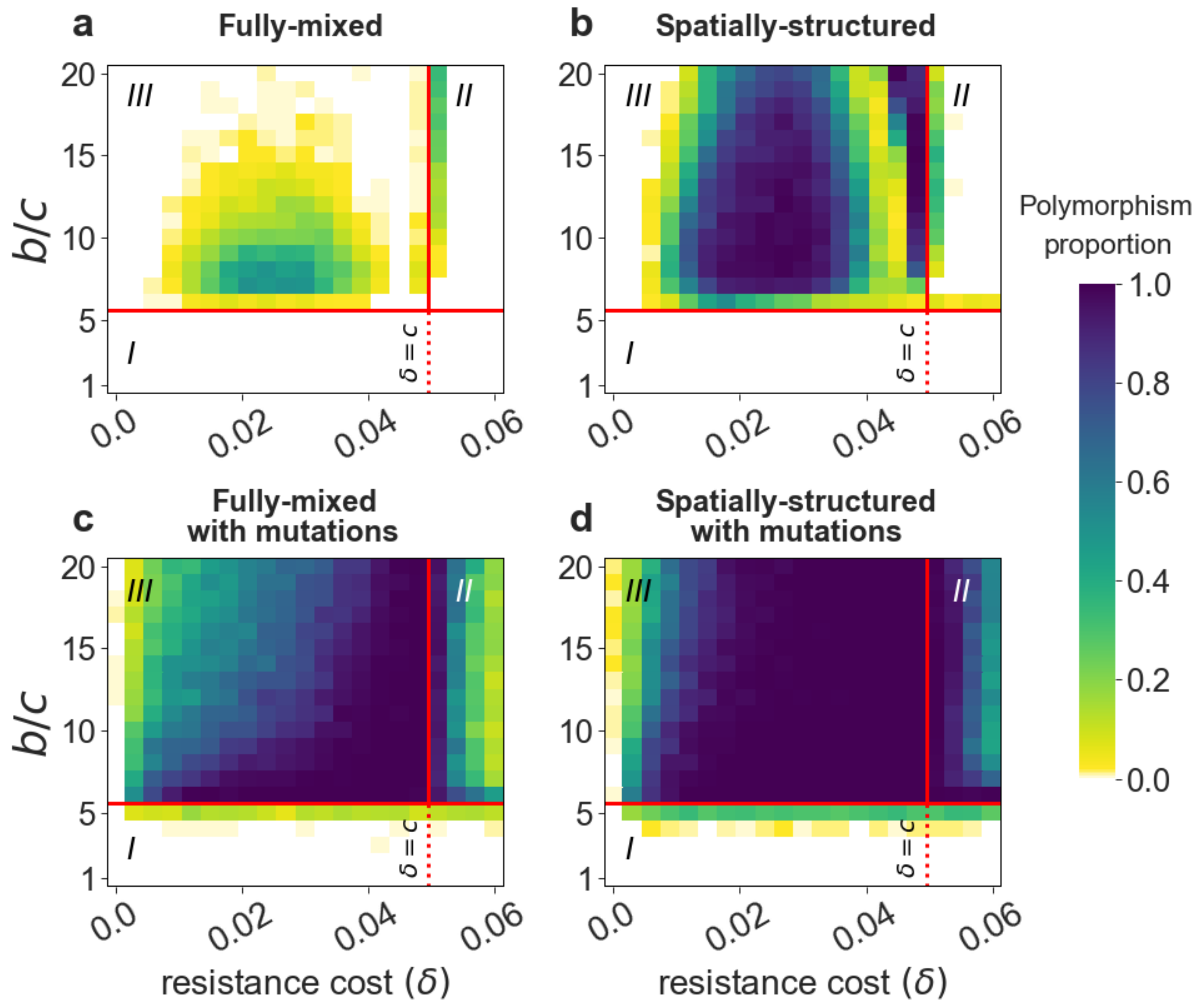

Figure S7. Intermediate expected proportions of cooperation are largely due to polymorphism. We plot (color coded) the proportion of simulations that ended while polymorphism is still maintained, namely reached 5000 generations with intermediate proportion of cooperators – above 0.03 and below 0.97. We can see that although polymorphism is rare in fully-mixed populations without mutations, polymorphism dominates the dynamics in spatially-structured population and in populations with mutations. This figure is based on the simulations presented in figure 4 in the main text. The color of each site represents the proportion (out of 200) of stochastic simulation runs ending in polymorphism. $T_\beta = 0.25, T_\alpha = 0.9T_\beta, c = 0.05, \mu = 10^{-4}$.

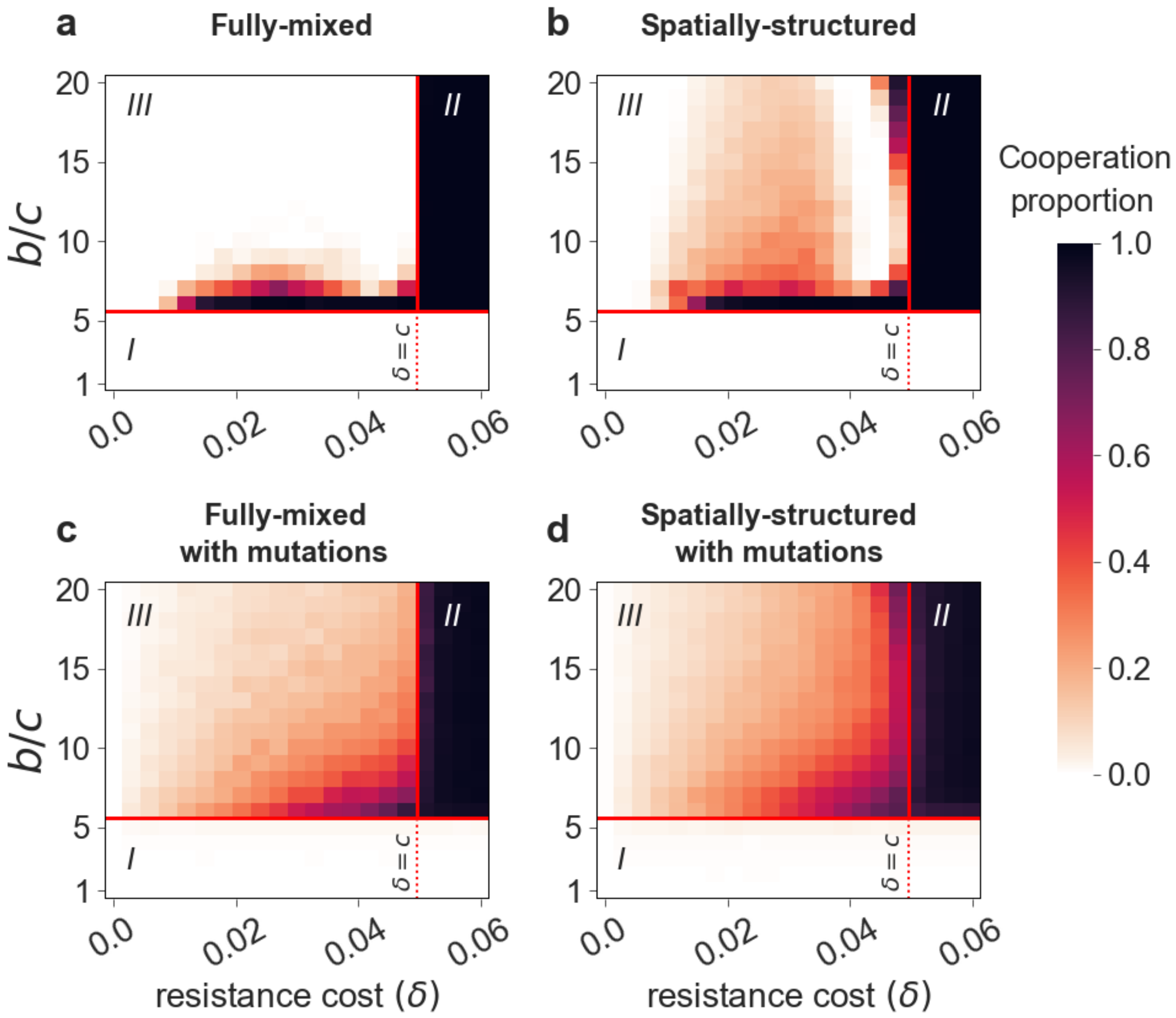

Figure S8. Maintenance of microbe-induced cooperation: robustness to stopping criteria. This figure is similar to Figure 4 in the main text except for the stopping condition. In this figure we show the proportion of cooperators after up to 10,000 generations (see SI4.1 for stopping criteria). The color of each site represents the average of 100 stochastic simulation runs. It can be seen that the results are very similar to the ones presented in Figure 4 in the main text, except for panel **(a)**, where the range of parameters allowing the maintenance of cooperation is narrower here. All simulations were initialized with equal proportions of the four host types. The horizontal red lines represent the analytic b/c threshold shown in inequality (2.1) in the main text.

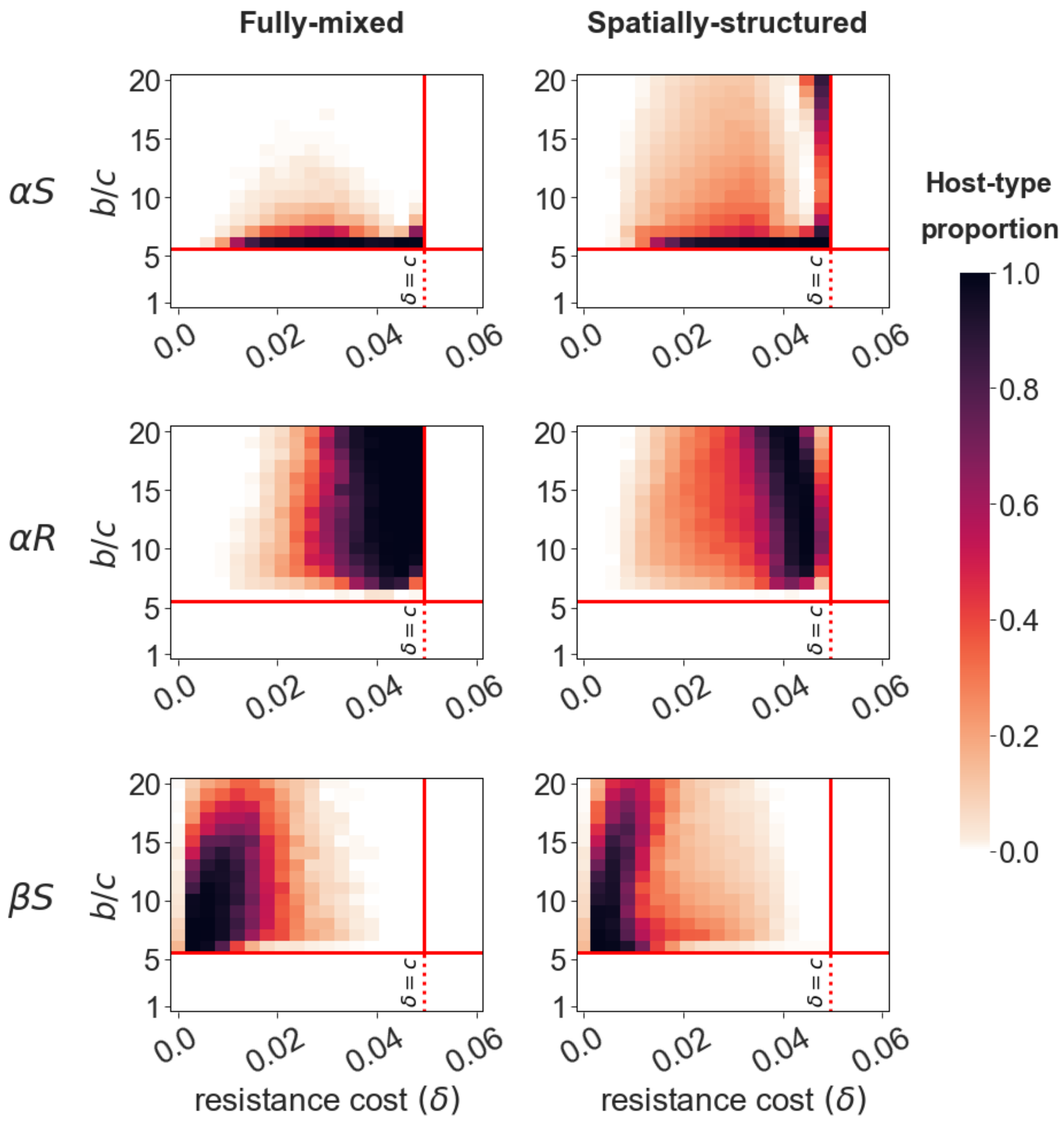

Figure S9. The expected proportion of cooperators is not monotonic in δ, in the absence of mutations. We plot (color coded) the expected proportion of three host types ($\alpha S, \alpha R, \beta S$; each in a different row), in two types of simulations (fully-mixed and spatially-structured; each in a different column). As explained in the **Stochastic models** section in the main text, when δ gets close to c, αR hosts tend to reach fixation, putting an end to the oscillations (2nd row, black areas). Similarly, when δ gets close to $0, \beta S$ hosts tend to reach fixation (3rd row, black areas). The figure is based on the same simulation runs as figure 4a,b in the main text. The horizontal red lines represent the analytic b/c threshold shown in inequality (2.1) in the main text.

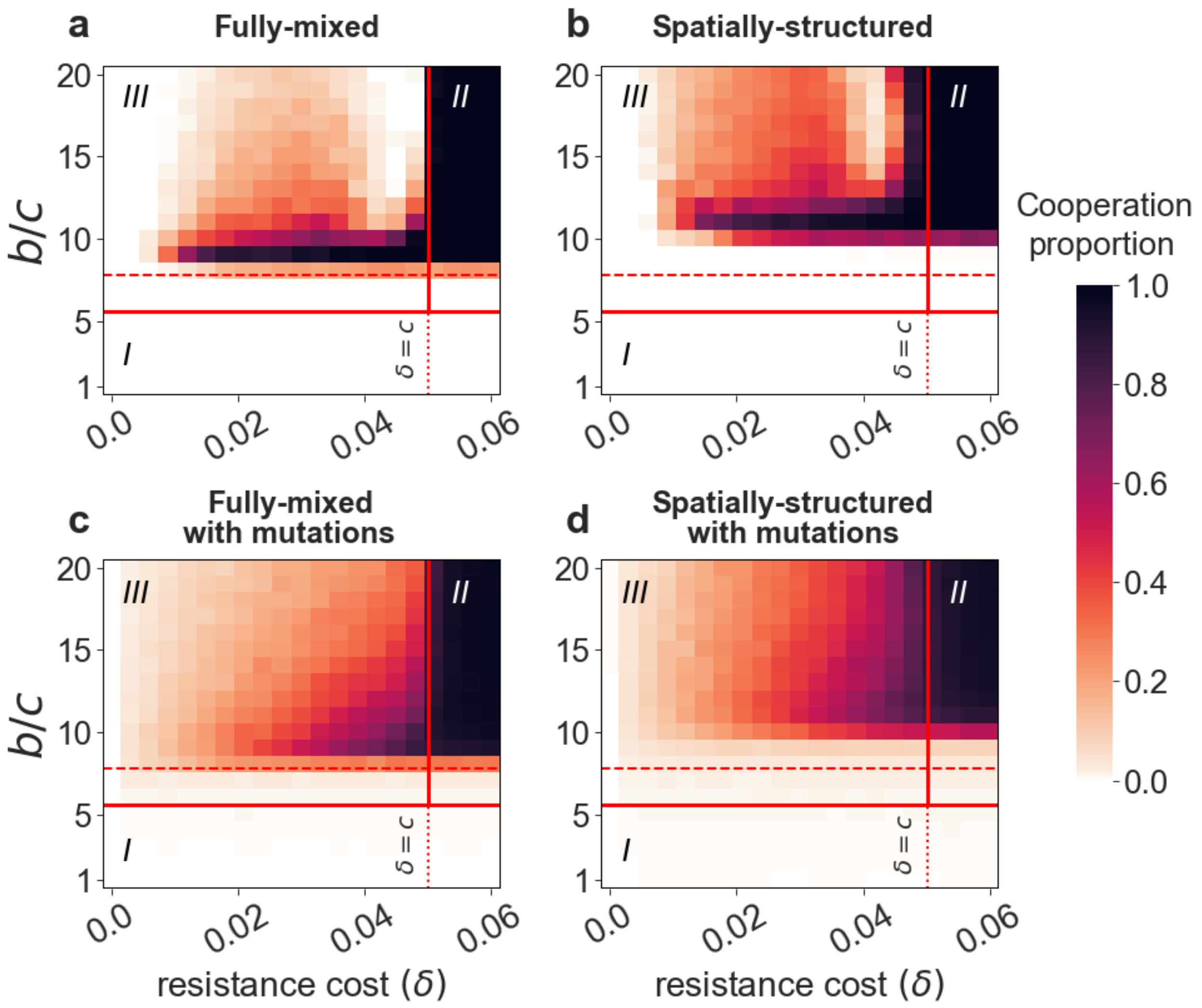

Figure S10. Maintenance of microbe-induced cooperation: robustness to imperfect vertical transmission. We plot the proportion of cooperators in simulations where the vertical transmission rate of both microbes is 0.5 ($\rho = 0.5$). The color of each site represents the average of 100 stochastic simulation runs. We find that cooperation can evolve even under imperfect vertical transmission, though the required b/c value is higher. We also note that in fully-mixed populations, the b/c threshold for the evolution of microbe-induced cooperation fits the analytic condition obtained in [Lewin-Epstein et. al. 2017; see also SI] (see red dashed horizontal line; the solid red horizontal line represents the b/c threshold in populations with $\rho = 1$). This threshold does not fit to the results of spatially-structured populations. $T_\beta = 0.25, T_\alpha = T_\beta \cdot 0.9, c = 0.05, N = 10{,}000, \mu = 10^{-4}$, one interaction per generation. All simulations were initialized with equal proportions of the four host types.

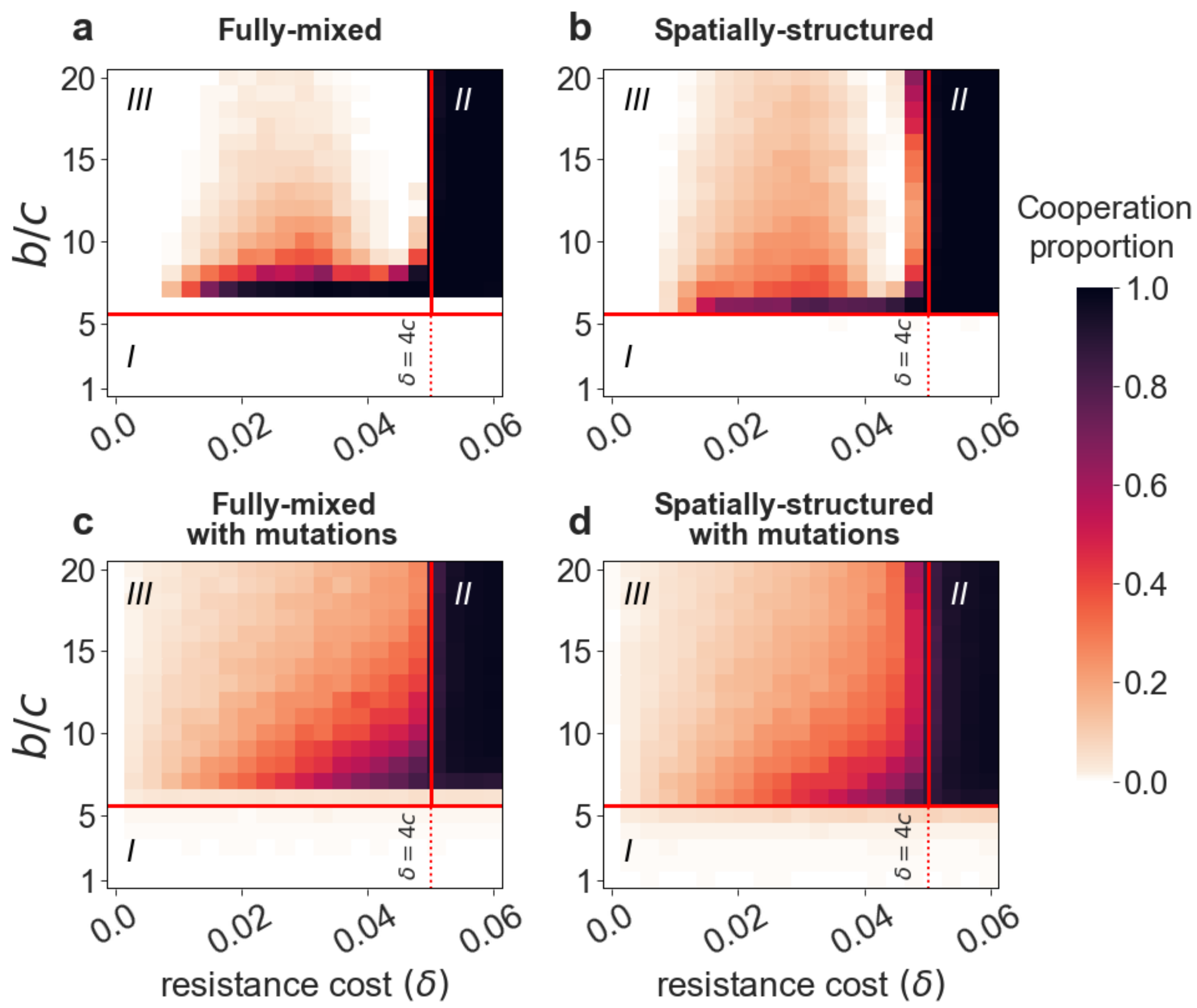

Figure S11. Maintenance of microbe-induced cooperation: robustness to multiple interactions per generation. We here show results of simulations with four interactions per host per generation (exactly four in the fully-mixed population; expected number of interactions is four in the spatially-structured population). The color of each site represents the average of 100 stochastic simulation runs. The horizontal red lines represent the analytic b/c threshold shown in inequality (2.1) in the main text. For comparison we normalize the costs and the transmission probabilities by setting $c = 0.05/4 = 0.0125$ and $T_\alpha = T_\beta \cdot \sqrt[4]{0.9}$. When using these parameters we obtained very similar results to those presented in figure 4 in the main text. Other parameters are: $T_\beta = 0.25,\ N = 10{,}000, \mu = 10^{-4}$. All simulations were initialized with equal proportions of the four host types.

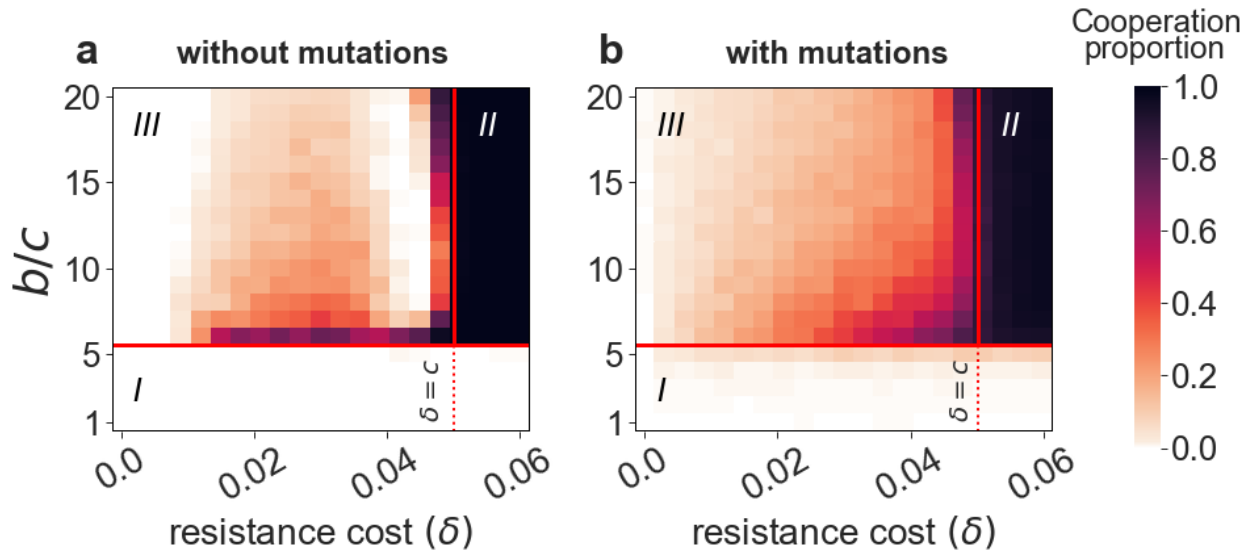

Figure S12. Maintenance of microbe-induced cooperation: robustness to multiple interactions per generation where fitness is normalized by the number of interactions. We here show results of simulations of spatially-structured populations with expectancy of four interactions per host per generation. The color of each site represents the average of 50 stochastic simulation runs. The horizontal red lines represent the analytic b/c threshold shown in inequality (2.1) in the main text. We normalized the fitness of each host by the number of interactions it participated in, so that the fitness of each host is: $1 - I_R \cdot \delta + \frac{b \cdot n_b - c \cdot n_c}{n_i}$ ($I_R = 1$ if the host carries allele R, and 0 otherwise; n_b is the number of times the host interacted with a cooperator; n_c is the number of interactions in which the host cooperated; n_i is the total number of interactions the host participated in). We also normalized the transmission probabilities by setting $T_\alpha = T_\beta \cdot \sqrt[4]{0.9}$. We obtained very similar results to those presented in figure S11 (panels b and d). Other parameters are: $c = 0.05, T_\beta = 0.25,\ N = 10{,}000, \mu = 10^{-4}$. All simulations were initialized with equal proportions of the four host types.
